# Rapid on-site methanol screening in distilled spirits via BME688 MOX sensor array and machine learning

**DOI:** 10.1038/s41598-026-49507-x

**Published:** 2026-04-25

**Authors:** İsmail Hakkı Parlak, Mehmet Milli, Nursel Söylemez Milli, Ferhat Demiray, Semra Turan

**Affiliations:** 1 Department of Computer Engineering, Bolu Abant Izzet Baysal University, Bolu, Turkey; 2 Scientific, Industrial and Technological Application and Research Centre, Bolu Abant Izzet Baysal University, Bolu, Turkey; 3 Department of Food Engineering, Bolu Abant Izzet Baysal University, Bolu, Turkey

**Keywords:** Methanol detection, Distilled alcoholic beverages, Gas sensor array, Machine learning, Electronic nose, Food safety, Chemistry, Engineering, Mathematics and computing

## Abstract

Methanol contamination in distilled beverages remains a serious public health risk, particularly in the context of illicit or poorly controlled production. Although confirmatory determination of methanol is routinely performed using laboratory-based techniques such as gas chromatography, spectrophotometry, and enzymatic assays, these approaches are often impractical for rapid screening due to infrastructure, expertise, time, and cost constraints. This study proposes a portable, fast and low-cost screening framework that integrates the Bosch BME688 metal-oxide (MOX) sensor array with machine-learning models to detect methanol-related patterns in mixtures at varying ratios. Measurements in experiments were conducted on controlled ethanol-methanol mixtures and extended to real beverage matrices (Raki- and Whisky-based samples) to better reflect practical conditions where matrix effects may confound detection. After generating the raw dataset, the first preprocessing step involved denoising the multi-sensor, multi-step heater signals. The data were temporally aligned, a logarithmic transformation was applied, and standardisation was ensured. Models have been constructed using multiple heater profiles and a series of classification and regression algorithms to evaluate both categorical discrimination and quantitative prediction capabilities. The results demonstrate that MOX sensor responses, when paired with appropriate preprocessing and learning models, provide a viable basis for rapid, in-situ methanol screening in distilled beverages, supporting early warning and prioritisation prior to confirmatory laboratory analysis. Further studies are planned to expand the range of commercially consumed beverages and to evaluate the study’s generalizability and limitations beyond controlled laboratory settings.

**Supplementary Information:**

The online version contains supplementary material available at 10.1038/s41598-026-49507-x.

## Introduction

Alcoholic beverages have a large consumer base worldwide and are an established component of daily life, social interaction, and cultural habits in many societies^1–4^. However, distilled alcoholic beverages are known to contain methanol, generally due to improper production conditions^5^. This highly toxic compound can cause serious health problems, including acute intoxication, irreversible vision loss, multiple organ damage, severe central nervous system effects, and death^6,7^. The presence of methanol in potable alcohol is typically associated with uncontrolled or poorly managed production processes, such as improper fermentation, inadequate distillation control, or contamination^8^.

Today, methanol determination in distilled alcohol is primarily achieved using traditional laboratory-based analytical methods. Among these, gas chromatography (GC), particularly GC-FID and GC-MS configurations, is considered the reference method owing to its high sensitivity and selectivity^9^, colorimetric–spectrophotometric^10^, and enzymatic approaches^11^. Widely used in regulatory and validation analyses, GC-based techniques allow for the quantitative and reliable determination of methanol^12^. In addition, spectrophotometric approaches — particularly methods based on chromotropic acid-based colourimetric reactions — provide quantitative evaluation by measuring the absorbance of the coloured complex formed upon chemical conversion of methanol^13^. Furthermore, enzymatic methods have been reported in the literature; these approaches generally rely on the enzymatic conversion of methanol via enzymes such as alcohol oxidase or methanol dehydrogenase^14^. Although these classical methods offer high analytical accuracy and reproducibility, they are not well suited to rapid, on-site, or mobile applications due to their need for controlled laboratory environments, specialised equipment, and expert personnel. This constitutes a significant limitation in situations that require timely risk assessment during field inspections and production phases.

Given the operational limitations of current analytical methods, a complementary screening layer is needed to enable rapid, cost-effective, and expert-independent detection of methanol under production and field conditions. This approach should not completely replace confirmatory laboratory methods but rather serve as a screening layer for early warning and preliminary risk assessment. Such an early-warning layer can support timely intervention and decision-making by enabling rapid identification of potential risks, prioritising suspicious samples for confirmatory analysis, and improving resource utilisation.

To address this gap, a significant portion of recent studies on rapid methanol screening have focused on gas sensors (electronic noses) and pattern-recognition-based approaches^15^. These systems aim not at the direct and selective measurement of a single analyte, but rather at classifying or quantitatively predicting the multivariate response patterns generated by volatile components in the sample’s headspace using different sensor systems. Machine learning methods such as Support Vector Machine, k-NN, and Artificial Neural Networks are therefore widely used to interpret sensor responses, enabling inference of methanol presence and level from multiple sensor signals^16,17^. In this context, metal-oxide (MOX) sensors are attracting particular attention for field-oriented applications due to their advantages, including low cost, rapid response, and portability^18^. A comparative overview of conventional analytical methods and recent sensor-based methanol detection studies is presented in Table 1.

**Table 1:** Quantitative comparison of the proposed BME688 metal oxide semiconductor (MOS) sensor array and machine learning framework with laboratory reference methods (GC-MS), recent sensor-based methanol detection studies, and a commercial portable organic vapour detector across key performance dimensions: detection type, screening time, estimated cost, power consumption, portability, limit of detection (LOD), operational complexity, real-sample validation, and classification/regression accuracy.

Study / Method	Technology	Detection Type	Detection Time	Estimated Cost	Power Consumption	Portability	LOD	Operating Complexity	Real Samples	Accuracy
GC-MS^9^ (Analytical reference)	Laboratory GC-MS	Quantitative	30–120 min (incl. sample prep)	$50k–$200k+	1–3 kW	No	~ 0.01–1 µg/mL	High	Yes	Very high
Hayasaka et al. (2020)^17^	Graphene FET + ML	Classification	~Minutes per scan	Low–moderate	Very low – room temp.	No	Not reported	High	No	High
Liu et al. 18(2021)^18^	MOS sensor array + ANN	Quantitative, Classification	~Minutes (30 s modulation period)	Moderate	~ 1–5 W	Semi-portable	< 400 mg/L (regulatory)	Medium	Yes, liquor	> 92%
Tonezzer et al. (2022)^16^	SnO₂-based MOX + ML	Quantitative, Classification	~Minutes	Low–moderate	~ 0.5–2 W	Semi-portable	~ 32 ppb (related study)	Medium	No	100%
Tozlu (2024)^15^	11× MQ sensors + ML	Classification	~ 40 s/recording (warm-up: 24 h+)	Low (<$50, MQ sensors)	~ 5–10 W (×11 MQ, ~ 800 mW each)	Semi-portable	Not reported	Medium	No	85.88%
Dräger PAC 8000 OV (Commercial portable)	Electrochemical / OV sensor	Quantitative (alarm-based)	< 30 s (T90 response)	~$400–$700	Battery-operated (< 0.1 W typical)	Yes	~ 1–2 ppm (range: 0–200 ppm)	Low	N/A	Alarm threshold only;
Proposed Study (BME688 + ML)	BME688 MOX sensor array + ML	Quantitative, Classification	~ 30 min/sample	~ 75 $	~ 31.2 mW (8× BME688 @ 3.9 mA, 3.3 V)	Yes	1.5% (v/v)	Low	Yes, raki/whisky	99%–100%

Among the studies in Table 1, Hayasaka et al. reported that the gate voltage was swept from − 40 to + 40 V at 2 V/s per scan, although the exact per-sample measurement duration was not specified. The same study described the device as low-cost and low-power, but no quantitative values were provided. Liu et al. employed a custom benchtop electronic nose system; however, exact cost and power data were not reported and were only estimated based on an STMicroelectronics STM32 microcontroller and MOX heater loads. They also referenced the methanol regulatory limit for Chinese liquor (< 400 mg/L), but no formal limit of detection was provided. Tonezzer et al. used a custom nanosensor platform, but the exact measurement duration, cost, and power consumption were likewise not reported. The reported LOD of 32 ppb was derived from a related study on SnO₂ nanowires by the same research group.

As shown in Table 1, conventional methods such as Gas Chromatography–Mass Spectrometry (GC-MS) provide very high analytical accuracy but lack portability. In contrast, recent sensor-based approaches offer faster and more flexible detection with lower operational complexity; however, many studies are limited to classification tasks, rely on controlled laboratory conditions, or lack validation in real beverage matrices.

The proposed Bosch BME688-based system distinguishes itself by combining classification and quantitative estimation with high accuracy, while being evaluated on real samples and designed with portability in mind. In addition, its compact architecture and low-power sensing capabilities support field-deployment scenarios. This positioning highlights its potential as a practical and deployable screening tool for methanol detection.

Despite these promising developments, several limitations remain in the literature. Many studies evaluate pure alcohols under controlled conditions, without adequately accounting for product-specific aroma effects on VOC profiles in real beverage matrices. Moreover, variability in the matrix across beverage types may substantially influence sensor responses and model generalisability. In addition, classification-focused approaches often overlook quantitative estimation and practical measurement protocols for rapid on‑site screening.

The present study aims to develop a rapid on-site methanol screening framework for distilled alcoholic beverages by integrating an array of BME688 metal-oxide (MOX) gas sensors^19^ with machine-learning and regression models. This approach seeks to exploit methanol-related volatile patterns to enable both effective classification and quantitative estimation. The results demonstrate the potential of this framework as a portable and cost-effective screening tool that can be integrated into production monitoring and field inspection workflows, while laying the groundwork for future validation and broader generalisation studies.

### BME688 sensor, specification, and capabilities

In this study, which aims to determine the methanol content in distilled alcoholic beverages, headspace VOC measurements were performed on the samples using a Bosch BME688 sensor. The Bosch BME688 is a miniaturised, multi-parameter environmental sensor that integrates a metal-oxide-semiconductor-based gas-sensing element with temperature, relative humidity, and barometric pressure sensors in a single package.

The BME688 sensor was considered not as a detector that directly and selectively measures a single compound, but rather as a chemical-fingerprint transducer that produces a reproducible response pattern to the VOC mixture originating from the distilled-beverage matrix. In addition, owing to its small size, low power consumption, and capability to generate high-sampling-rate time-series signals under controllable heater programs, the sensor offers practical advantages for rapid screening-oriented sensing platforms.

### Usage areas of the BME688 sensor

The BME688 sensor’s ability to provide sensitive measurements and respond to a broad spectrum of volatile compounds supports its use across diverse application areas. Accordingly, it can be customised for domains where environmental parameters, particularly temperature, humidity, and VOC-related signals, are critical. In the present study, this versatility was leveraged to adapt the BME688 for the analysis of distilled alcoholic beverages, aiming to capture reproducible response patterns amid complex VOC backgrounds and to improve discrimination and quantitative estimation of methanol in the presence of matrix-derived volatiles.

Recently, the BME688 sensor has been used in numerous application scenarios in the literature. The most common scenarios include indoor and outdoor air quality monitoring^20,21^, food classifications^22^, food freshness assessment^23^, and food adulteration detection^24,25^. Additionally, safety-focused scenarios include early fire detection in forestry and agricultural areas^26^, anomaly detection in critical areas and laboratory environments^27^, early detection of environmental risks in military facilities and field conditions^28^, bad-smell and leak-detection monitoring^29^. Furthermore, it has been observed in areas such as disease diagnosis, monitoring, and microbial activity detection^30^. Moreover, the sensor has shown potential for use in other applications involving Volatile Organic Compounds (VOCs), volatile sulphur compounds (VSCs), and other gases such as carbon monoxide and hydrogen.

Beyond these established applications, the BME688 sensor holds significant potential for future deployment in portable and real-time monitoring systems. In particular, its integration with embedded platforms and machine-learning algorithms may enable on-site, rapid screening tools that do not require complex laboratory infrastructure. The ability to detect subtle variations in VOC patterns further suggests its applicability in early-stage anomaly detection and quality assurance processes.

## Materials and methods

### Sensor matrix and sensor matrix configuration

Instead of using a single sensor to measure environmental conditions and detect methanol in the mixture, a matrix of 8 BME688 sensors was employed^31^. There are many uncontrollable parameters affecting the measurement environment. Therefore, recreating the exact same experimental conditions at different times is unrealistic. For this reason, using multiple sensors simultaneously is crucial for testing different profile settings in the same experimental environment. Furthermore, increasing the volume of data collected per unit time also improves the accuracy of artificial intelligence models.

**Fig. 1:**
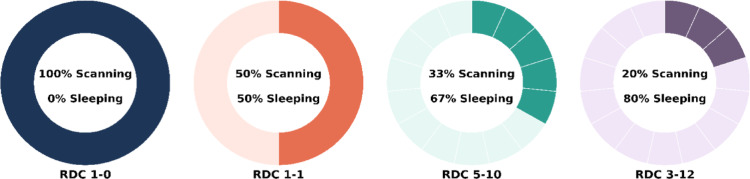
Different RDC profiles can be used across a range of application scenarios, depending on power consumption and accuracy requirements. (adapted from^32^.

There are two important configurations that determine the sensor’s measurement capabilities and affect the characteristics of the generated dataset. The first is the Real Duty Cycle (RDC) profile, which governs the sensor’s scanning and sleep cycles. The RDC profile can save up to 80% of power, particularly in scenarios where sensor nodes are externally powered and far from the city grid, thereby extending their lifespan. Figure 1 shows some RDC profiles that can be configured for BME 688 sensors in different application scenarios.

In this study, because the sensor nodes were powered by the city grid and artificial intelligence models required collecting more data per unit time to make more stable decisions, the nodes were never put to sleep during data collection. The nodes were operated in continuous scanning mode using the RDC1-0 profile.

In terms of power consumption, the BME688 sensor draws approximately 3.9 mA during standard gas scan mode^33^. When lower-power RDC profiles are employed, consumption can be reduced to approximately 0.9 mA in low-power mode or as low as 90 µA in ultra-low-power mode, as specified in the Bosch Sensortec datasheet. These figures are well within the operating envelope of battery-powered, portable devices, confirming the sensor’s suitability for field-deployable applications. Regarding computational requirements, the machine learning models employed in this study — particularly the SVM classifier and lightweight regression models — have low inference-time complexity and small serialised footprints.

To evaluate the feasibility of embedded deployment, the trained SVM model was converted to C code using micromlgen for deployment on ESP32-class microcontrollers. The resulting implementation required approximately 9 KB of flash memory and less than 1 KB of RAM. The model operates on 20 input features derived from a sensor pair (2 sensors × 10 heater steps), enabling lightweight inference suitable for microcontroller-based systems. Based on the computational complexity and model size, sub-millisecond inference time is expected on ESP32 hardware, confirming feasibility for embedded deployment and low-latency on-device inference.

In addition, power consumption was estimated for an ESP32-based system interfaced with two BME688 sensors. The total system power consumption during active operation is estimated to be approximately 0.25–0.30 W. Using a 2000 mAh battery, continuous operation of approximately 20–25 h is achievable, demonstrating suitability for portable and battery-powered applications. These results provide preliminary verification that the proposed sensing and machine learning framework can be deployed on low-power embedded platforms.

Another parameter that determines the sensor’s operating status is the Heater Profile (HP). In metal-oxide semiconductor (MOX) sensors, gas interaction occurs at the sensor surface. VOC molecules adhere to (adsorption) or detach (desorption) from the sensor surface. Increasing or decreasing the sensor’s operating temperature (temperature modulation) alters the relative rates and balance of these two processes. In MOX sensors, modulation of the operating temperature significantly affects the dynamic character of the sensor response by altering VOC adsorption-desorption kinetics and surface reactions.

**Fig. 2:**
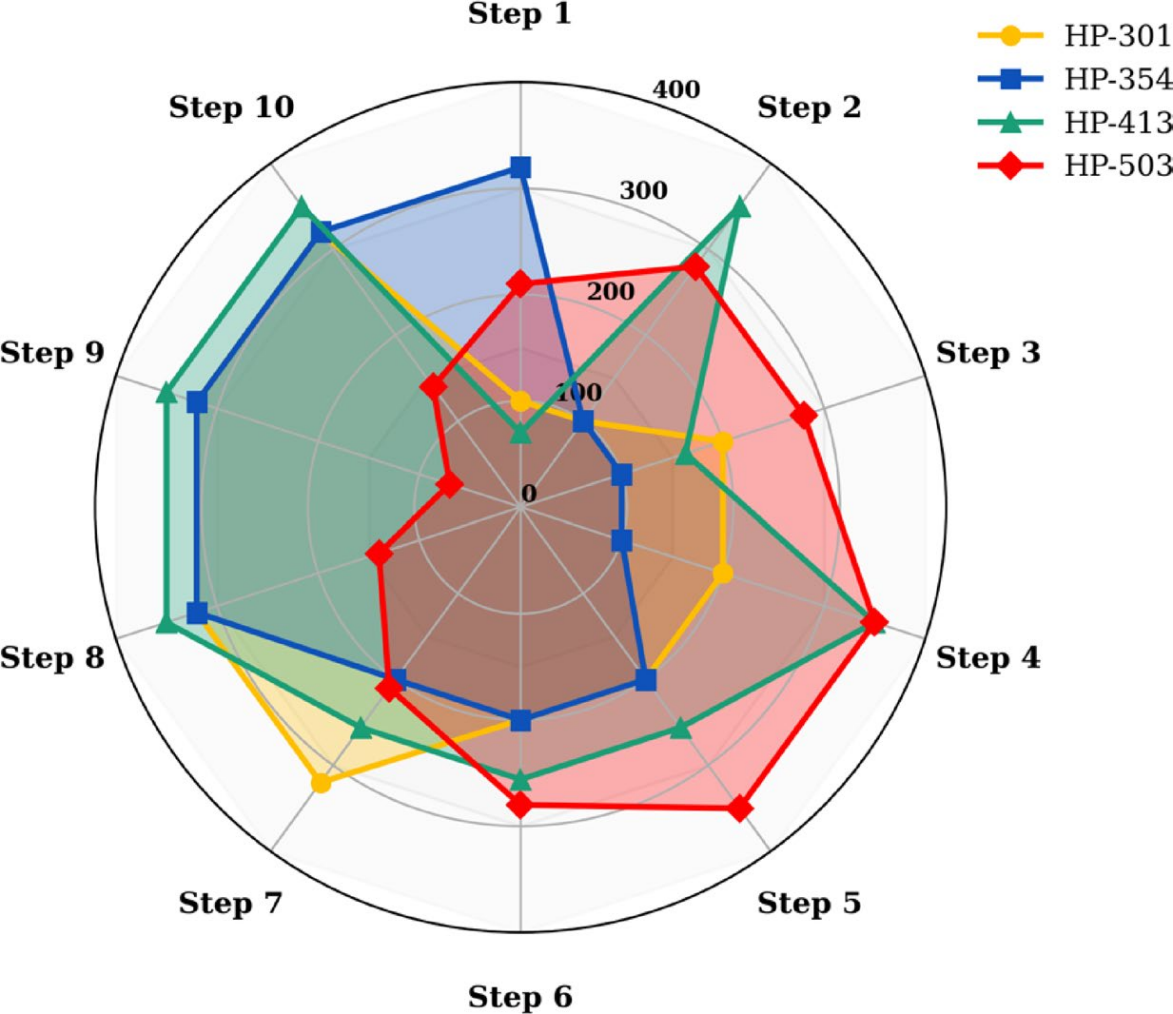
Temperature modulation graphs for four different temperature modulation profiles used to configure the BME688 development kit. Each heater profile (HP) modulates the temperature in a specific manner to measure the resistance of the ambient gas mixture. (adapted from^32^.

In this study, four distinct temperature modulations were applied simultaneously to sensors. This enabled identification of the HP profiles most effective for detecting methanol in potable alcohol. Figure 2 shows the HP profiles used in this study (HP-301, HP-354, HP-413, HP-503).

**Fig. 3:**
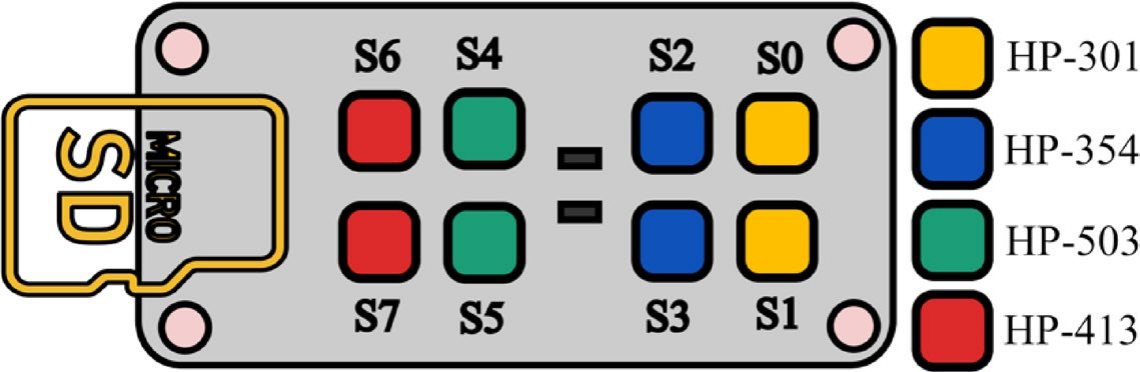
BME688 development kit components and Heater Profile (HP) configurations for the sensor pairs in the sensor kit. S 0, 1 denotes the first sensor pair, S 2, 3 denotes the second sensor pair, and so on.

The eight sensors in the sensor matrix are grouped in pairs, with each pair configured using a different HP profile. Figure 3 shows the components of the BME688 development kit and the HP profiles configured for each sensor pair in the matrix.

This strategy enables simultaneous measurements of 4 different HPs in the same experimental setup and verifies the electrical resistance measured by one sensor against another loaded with the same HP.

### Experimental setup

In this study, data were collected in three stages. In the first stage, a measurement setup was prepared to test the sensor’s capability to detect pure alcohol, excluding product-specific odour and aroma effects of commonly consumed alcoholic beverages. This initial phase provided a controlled baseline for evaluating sensor response solely to alcohol-derived VOC patterns.

There is no standard value for dangerous methanol levels in potable alcohol^7^. While some limit values ​​are reported in the literature, these can vary from person to person^6,8,34^. Consequently, because this study is primarily an analytical screening and classification study rather than a chemical-dose study, methanol concentrations well below legally hazardous limits were used to test sensor and machine-learning capabilities. The classes were created with this conceptual framework in mind.

To enable the sensor to recognise VOC fingerprints in alcohol, samples were prepared by mixing commercially available ethanol (Eth) and methanol (Met) in different ratios. A total of 6 mixture samples, prepared at different ratios, were left at room temperature for a while to allow homogenisation. The mixture samples used for measurement were prepared as follows: (1) Mixture 100% Eth. – 0% Met., (2) Mixture 97.5% Eth. – 2.5% Met., (3) Mixture 95% Eth. – 5% Met., (4) Mixture 90% Eth. – 10% Met., (5) Mixture 80% Eth. – 20% Met., and (6) Mixture 50% Eth. – 50% Met. In all samples, the total volume of the mixture was set at 200 ml.

After placing the 6 mixtures into different Erlenmeyer flasks, measurements began on December 12, 2025, at 09:00 and ended at approximately 15:00. Sensor Matrix 0 was used only to measure the samples in the flasks, while Sensor Matrix 1 was used to measure the empty flask. The sensors were operated at room temperature for approximately one hour to allow them to stabilise. This stabilisation step was critical for minimising baseline drift and ensuring repeatable sensor responses.

After the sensors stabilised, Sensor Matrix 0 was positioned in the first Erlenmeyer flask and measurements were taken for 30 min. After each sample measurement, to allow any remaining VOCs from the previous sample to dissolve on the sensor membranes, the sensors were allowed to measure at room temperature for 20 min. Subsequently, measurements were taken from each bottle for the same duration, using the same procedures.

In the second phase of the data collection process, similar experimental mixtures were used, this time with raki, a commercially consumed alcoholic beverage in Turkey. This data collection process began on December 22, 2025, at 09:00 and ended at approximately 14:00. Data were collected using the same measurement procedures as in the previous phase.

Many commercially sold raki have an alcohol content between 40% and 50%^35–37^. To maintain the alcohol content, five grades were prepared by mixing ethanol, methanol, and pure water in varying proportions, each with 100 ml of pure raki. This approach allowed controlled variation in methanol content while preserving the overall characteristics of the alcohol matrix.

The mixture samples used for measurement were prepared as follows: (1) Mixture 100 ml raki − 55 ml water – 45 ml Eth. – 0 ml Met., (2) Mixture: 100 ml raki − 55 ml water − 42 ml Eth. – 3 ml Met.,, (3) Mixture 100 ml raki − 55 ml water – 37 ml Eth. – 8 ml Met., (4) Mixture 100 ml raki − 55 ml water – 35 ml Eth. – 10 ml Met., and (5) Mixture 100 ml raki − 55 ml water – 30 ml Eth. – 15 ml Met.

**Fig. 4:**
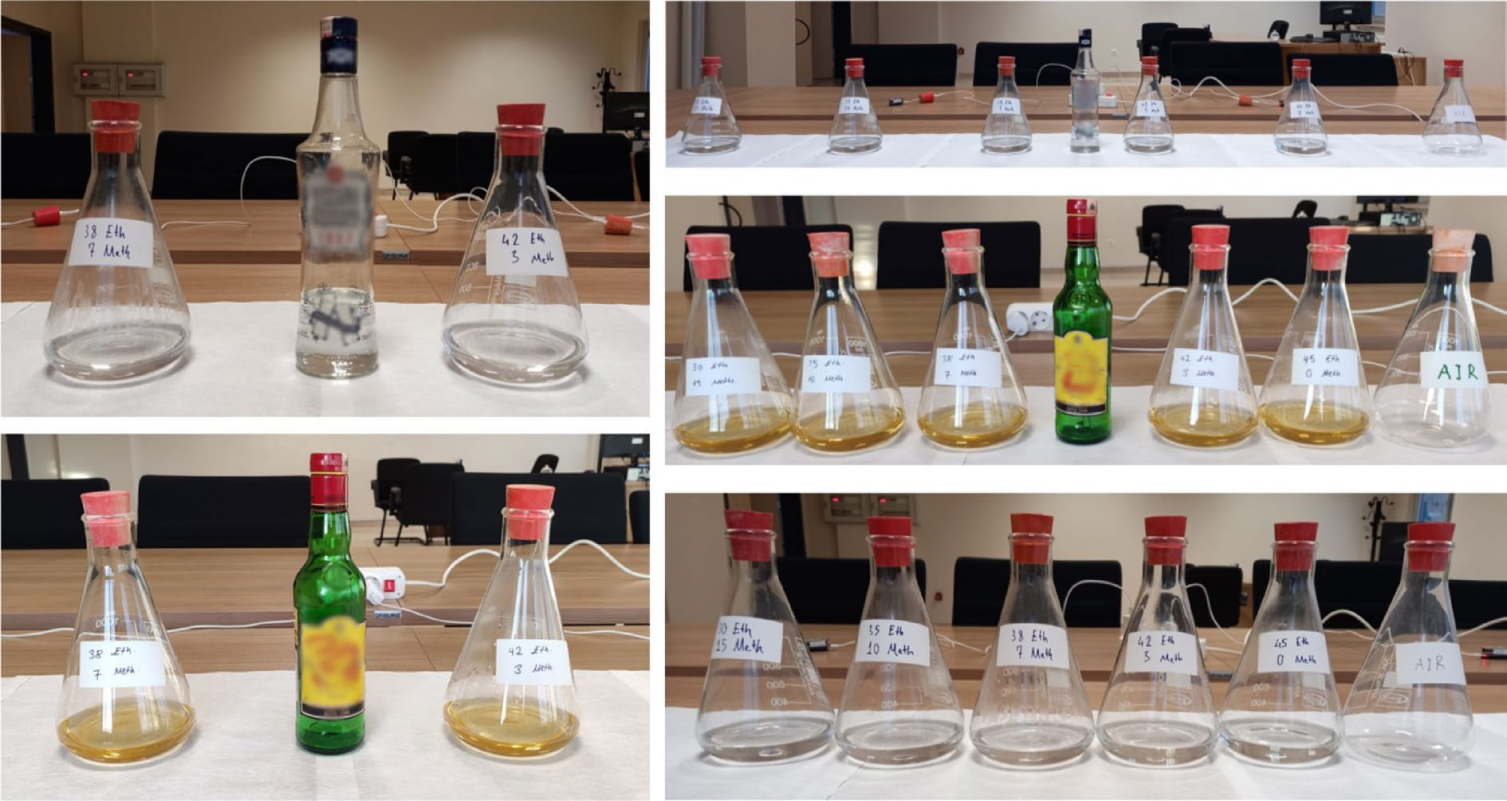
Experimental setup for detecting the presence and concentration of methanol in pure alcohol, raki, and whisky mixtures using the BME688 sensor.

The third phase of the data collection process was carried out with whiskey, another commonly consumed distilled alcohol frequently associated with methanol poisoning cases worldwide. All the mixtures used in the raki samples were applied to the Whiskey in the same proportions. Data collection, initiated with 5 samples created using different ratios of Whiskey, water, ethanol, and methanol, was conducted on December 24, 2025. Data collection began at 09:00 and continued until approximately 14:00. The experimental setup, prepared using mixtures of whisky, water, ethanol, and methanol, is shown in Fig. 4.

The lowest methanol concentration tested in both the raki and whisky matrices was 1.5% (v/v). For context, Paine and Dayan^34^ estimated a tolerable daily dose of 2 g methanol for an adult and derived an MTC of 2% (v/v) in a 40% ABV drink, while noting that the EU general limit of 0.4% (v/v) provides a considerably wider safety margin. The concentrations investigated in the present study, therefore, span a range of direct toxicological and regulatory significance. A formal analytical Limit of Detection (LOD) was not determined in this study, as the primary objective was classification and quantitative prediction across predefined concentration classes; determining an LOD remains an important direction for future work.

## Model selection

After data collection was complete and datasets were created, the data were pre-processed to improve the performance of regression and classification models. Once the data were ready for use, they were subjected to regression and classification algorithms commonly used in the literature, and the results were compared.

### Raw data and dataset

**Fig. 5:**
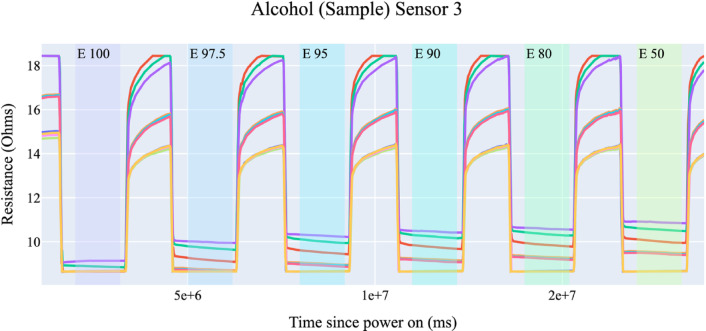
Raw data acquired from Sensor 3 during the pure alcohol experiment. Labels E100, E97.5, etc., indicate regions corresponding to measurements of ethanol–methanol mixtures. E95 denotes a 95% ethanol–5% methanol mixture.

As a result of the data collection process, three independent datasets were created: Alcohol, raki, and whisky datasets. Figure 5 shows the raw data from sensor number 3 in the Alcohol dataset of the sensor matrix. The classifications in this dataset are labelled by the ethanol concentration in the mixture.

As shown in Fig. 5, as the methanol concentration in the mixture increases, the gas composition’s resistance in the environment increases. If an imaginary vertical line is assumed from any point on the graph in Fig. 5, it can be said that this line forms a unique fingerprint of that moment on the time axis. The alcohol dataset comprises 8.812 data cycles. Each data cycle contains 10 data points. Therefore, this dataset comprises 88.120 data points.

**Fig. 6:**
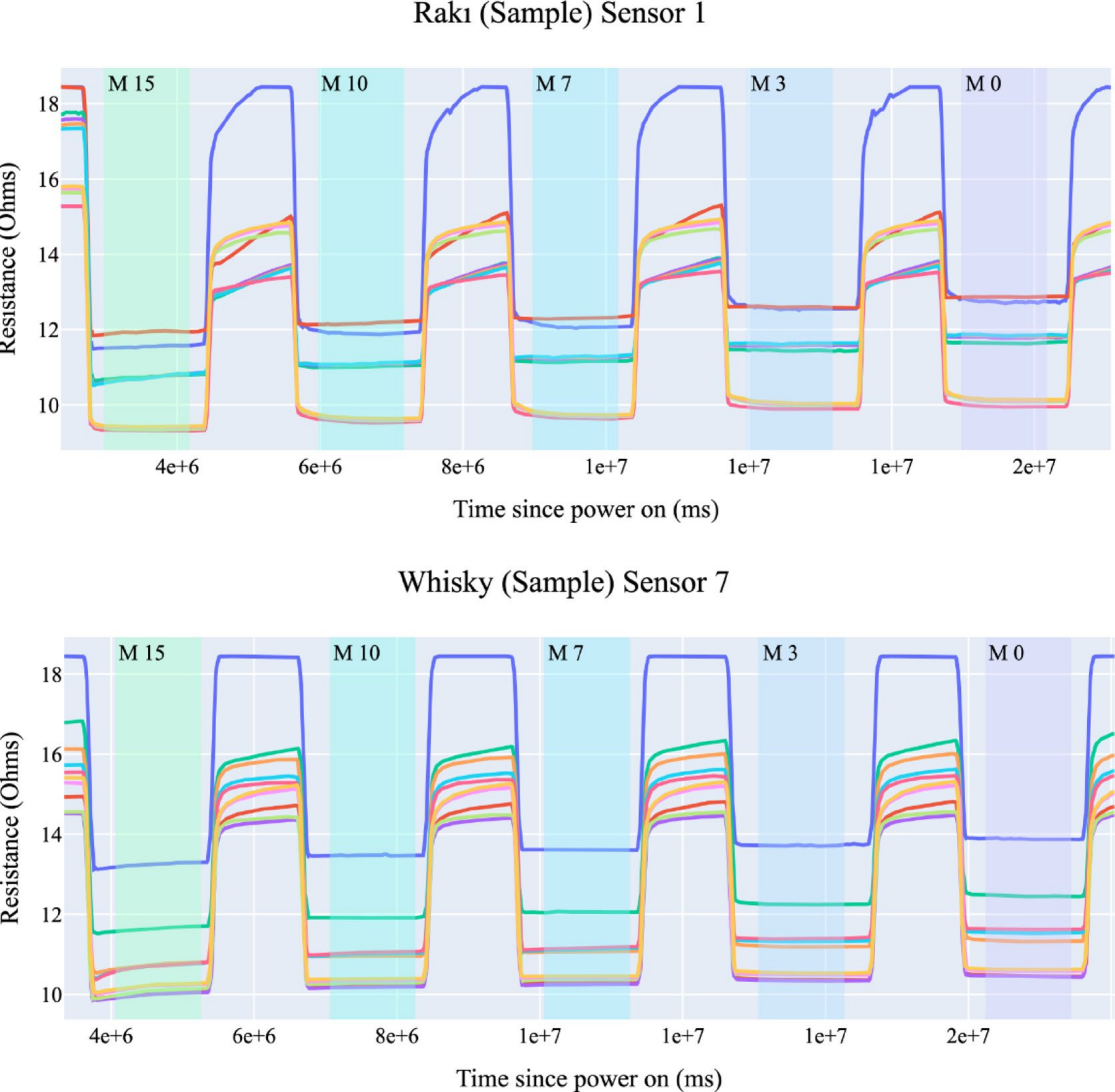
Raw data acquired from Sensor 1 during the raki experiment and from Sensor 7 during the whisky experiment, respectively. Labels M15, M10, etc., indicate regions corresponding to methanol-mixed samples. For example, M15 denotes the presence of 15 ml of methanol in the mixture.

The graph shows that the classes are evenly distributed in the dataset, ensuring proportional representation when building regression and classification models. The other datasets, raki and whisky, contain 7.851 and 8.306 data cycles, respectively. This corresponds to 78.510 and 83.060 data points, respectively. Figure 6 shows the classes in the raki and whisky datasets, along with the raw data collected by sensor 1 in the raki dataset and sensor 7 in the whisky dataset.

The names of the classes in the raki and whiskey datasets are derived from the millilitre-level values ​​of the methanol content in the mixture. As shown in Fig. 6, as in the Alcohol dataset, a positive correlation is observed between methanol in the mixture and the electrical resistance of the gas mixture measured by the sensors in both datasets.

### Data preprocessing

In this study, the output of the MOX sensors used as ambient measurements reflects the total electrical resistance of the VOCs in the mixture, rather than being specific to a single gas type. Therefore, in this study, which was conducted to detect the presence and level of methanol in drinkable alcohol, ethanol, which is dominant in distilled beverages, and product-specific aroma components can mask the methanol signal. Consequently, in the literature, preprocessing sensor data is widely recognised as a critical requirement for model performance and generalizability.

The gas sensor matrix data is saved as files with the .bmerawdata extension on an external SD card. This file type is based on the JavaScript Object Notation (JSON)^38^ format, which is readable by both humans and machines. Before proceeding with the preliminary data processing steps, the data in JSON format was imported into the software environment. For this purpose, JSON parsers were used, and the raw data was converted into Pandas DataFrame^39^ instances to facilitate further processing.

As previously noted, each sensor matrix comprises eight sensors, and each sensor completes a measurement period comprising 10 heating steps. Consequently, a single sensor matrix produces a time series of 8 × 10 = 80 gas-resistance per unit of time. As the first preprocessing step, each gas resistance time series of the data frame was denoised using a low-pass filter^40^. After this filtering process, a smoother time series was obtained by removing high-frequency components from the sensor’s electrical resistance signals.

In the second stage of the data preprocessing step, the classification of data in 3 datasets was performed. In the alcohol dataset, data were labelled by ethanol percentage in the mixture, whereas in the raki and whiskey datasets, classification was based on methanol content in millilitres. For example, in the alcohol dataset, a mixture containing 80% ethanol was labelled as “ethanol 80”. In contrast, in the raki and whiskey datasets, a mixture containing 3 millilitres of methanol was labelled as “methanol 3”, and similar labelling was applied to other concentrations.

**Fig. 7:**
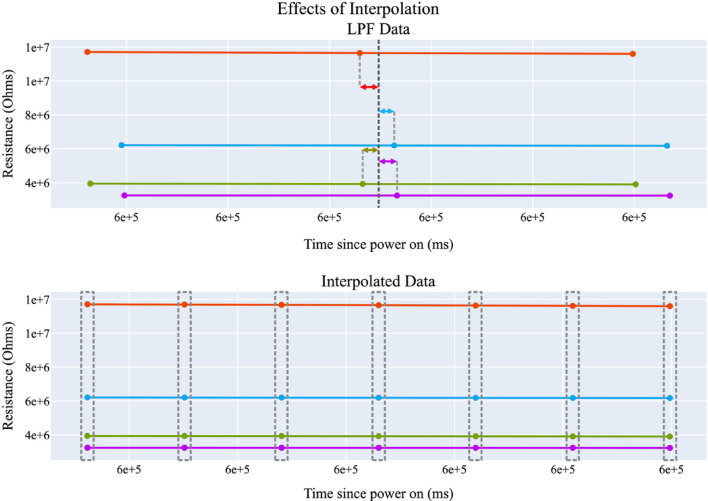
Effect of the interpolation function applied to the data. Non-interpolated samples are shown on the top graph. Interpolated and time-aligned samples are shown on the bottom graph.

In the third step of the data preprocessing, the data was interpolated using single-variable spline functions^41^. Interpolation makes the data continuous, enabling the creation of synthetic samples at the required time points. The creation of synthetic data significantly expands the dataset and improves the training of classification models. Furthermore, the interpolation method was used to temporarily align the data points. The effect of the interpolation function applied to the data is seen in Fig. 7.

The electrical resistance values ​​of the gas composition in the alcohol mixtures in all three datasets were observed to range from approximately 10^3^ to 10^9^ ohms. Visualising data with such large variance is difficult, and it can also suppress error functions associated with high-magnitude observations, thereby adversely affecting the stability and generalizability of both regression and classification models. To address high variance and heteroskedasticity, the data were first log-transformed. Then, standard scaling, a commonly used technique to improve the stability and convergence of machine learning training, was applied^42^.

### Model selection

Regression and classification models were created using three different datasets generated during the data collection phase. The purpose of the classification models is to distinguish among different alcohol mixtures based on their gas resistance values and to assign each sample to the previously defined class. The purpose of the regression models is to create a regression curve using cases with known measurement values ​​and to use this curve to predict cases with unknown measurement values.

This study utilised two different sensor matrices, each containing eight BME688 sensors capable of independent measurement. Sensor matrix 0 was used to measure the electrical resistance of methanol-ethanol mixtures prepared at different gas concentrations. Sensor matrix 1 was placed in an empty Erlenmeyer flask under the same ambient conditions and used to measure the electrical resistance of varying, uncontrollable environmental parameters throughout the measurement period. After data collection, the electrical resistance value obtained from sensor matrix 1 was subtracted from that of sensor matrix 0 to quantify the net effect of alcohol mixtures on the sensor output.

This procedure compensated for ambient gas composition, temperature, and humidity fluctuations by isolating the effect of alcohol mixtures from background environmental variability. Since both matrices comprise identical sensor types operated under the same heater profiles and exposed to the same ambient environment, any common-mode drift is effectively cancelled by this differential approach. All measurements were conducted within a single continuous session of approximately six hours; over this timeframe, long-term sensor aging and baseline drift are not expected to be significant factors, as confirmed by the stable sensor signals observed in Figs. 5 and 6. In other words, by identifying only the VOC values to be measured, machine learning approaches were able to produce more stable results. It should be noted that characterising long-term drift behaviour across multi-day or multi-week deployments remains an important direction for future work.

Both sensor matrices were configured with the same heater profiles: HP-301, HP-354, HP-503, and HP-413. These HP profiles were sequentially assigned to the four sensor pairs in each sensor. Due to differences in measurement configurations, each heater profile yielded a different resistance value. The contribution of each temperature-modulation to the sensor pairs to model performance was evaluated separately.

Decision tree (DT), Gaussian Naive Bayes (GNB), support vector machine (SVM), logistic regression (Log. R), and multilayer perceptron (MLP) classifiers, which are frequently used in the literature for data classification, were employed. Each developed model was trained using Stratified Group K-Fold cross-validation (StratifiedGroupKFold)^43^, a strategy that extends standard k-fold cross-validation by jointly enforcing two constraints: (1) each fold’s test set contains a representative proportion of all class labels, and (2) samples belonging to the same group are never split across training and test folds. For the raki and whisky datasets, each containing 500 samples (5 classes × 100 samples per class), samples were divided into 10 contiguous groups of 50 samples each, yielding 2 groups per class, and a 5-fold strategy was applied. For the pure alcohol dataset, which contained 600 samples (6 classes × 100 samples per class), samples were divided into 12 contiguous groups of 50 samples each, again yielding 2 groups per class, and a 6-fold strategy was applied. In all cases, each fold held out exactly one group per class as the test set, ensuring that the models were always evaluated on entirely unseen sample batches. This prevents data leakage that would otherwise arise from standard k-fold cross-validation, where samples from the same recording batch can appear in both training and test folds, leading to optimistically biased accuracy estimates. The accuracy scores were compared between different heater profiles and classification accuracy results are presented in Table 2.

For regression, linear regression (LR), gradient-boosting regressor (GBR), stochastic gradient descent regressor (SGDR), support vector regression (SVR), and Bayesian ridge regression (BR) models were trained. The dataset was split into 80% training and 20% testing subsets. Models were trained on the training data and evaluated on the test data. Mean absolute error (MAE) and $\:{R}^{2}$ scores were computed to assess regression performance. The MAE and $\:{R}^{2}$ results are reported in Tables 3 and 4, respectively.

## Results and discussion

Table 2 presents the classification accuracy scores of the evaluated algorithms across different heater profiles and alcohol mixture groups. As shown in the table, the support vector machine (SVM) model achieves perfect classification performance on raki and whisky data when configured with the HP-301 profile. For pure alcohol mixtures, HP-354 yields near-perfect accuracy results, followed closely by the HP-301 profile.

**Table 2:** Classification accuracy scores for Decision Tree (DT), Gaussian Naïve Bayes (GNB), Support Vector Machine (SVM), Logistic Regression (Log. R), and Multilayer Perceptron (MLP) classifiers across the selected heater profiles: HP-301, HP-354, HP-503, and HP-413.

	Heater Profile	DT	GNB	SVM	Log. R	MLP
Pure alc.	HP-301	0.768	0.885	0.983	0.750	0.782
	HP-354	0.855	1	1	0.780	0.917
	HP-503	0.902	0.910	0.988	0.860	0.903
	HP-413	0.958	0.673	0.960	0.745	0.807
Raki	HP-301	1	1	1	1	1
	HP-354	0.854	0.770	0.950	0.926	0.968
	HP-503	1	0.854	0.998	0.998	1
	HP-413	1	0.942	1	1	1
Whisky	HP-301	1	1	1	1	0.994
	HP-354	0.838	0.950	0.978	0.912	0.948
	HP-503	0.828	0.738	0.902	0.774	0.780
	HP-413	1	0.982	1	0.990	0.982

Table 3 presents the mean absolute error (MAE) scores of the regression models for predicting the methanol concentration in alcohol mixtures. The gradient boosting regressor (GBR) achieves a perfect MAE score of 0 when trained on data generated using the HP-301 or HP-503 heater profiles across all three alcohol mixture groups. Considering the R^2^ values ​​in Table 4, it is observed that the GBR model, which has the best performance in predicting methanol concentration in different alcohol types (pure alcohol, raki, and whisky datasets), is again the best, in parallel with the MAE results in Table 3.

**Table 3:** MAE scores of linear regression (LR), gradient boosting regressor (GBR), stochastic gradient descent regressor (SGDR), epsilon-support vector regression (SVR), and Bayesian ridge regression (BR) models across the selected heater profiles: HP-301, HP-354, HP-503, and HP-413. Lower MAE values indicate better performance.

	Heater Profile	LR	GBR	SGDR	SVR	BR
Pure alc.	HP-301	0.770	0	1.133	2.057	0.767
	HP-354	0.802	0.011	1.296	0.985	0.798
	HP-503	0.532	0	0.840	0.817	0.533
	HP-413	0.543	0.036	0.822	1.215	0.543
Raki	HP-301	0.126	0	0.407	0.099	0.126
	HP-354	0.219	0	0.463	0.111	0.220
	HP-503	0.130	0	0.337	0.098	0.130
	HP-413	0.176	0	0.383	0.127	0.175
Whisky	HP-301	0.192	0	0.377	0.095	0.193
	HP-354	0.164	0	0.417	0.086	0.163
	HP-503	0.125	0	0.274	0.097	0.124
	HP-413	0.099	0	0.283	0.109	0.098

When the results are interpreted in terms of temperature modulation profiles (HP), as seen in Tables 2 and 3, and 4 in the classification and regression models, it is thought that the BME688 gas sensor shows near-perfect performance for methanol detection and concentration in alcoholic beverages when configured with HP-301 or HP-503 heater profiles.

In conclusion, for the detection and classification of methanol in widely consumed potable distilled spirits, SVM-based models perform best, regardless of temperature modulation. Furthermore, for detecting methanol in pure alcohol, the best combination of temperature modulation and algorithm is achieved by models that integrate the SVM algorithm across all temperature-modulation profiles. To determine methanol concentration in pure alcohol, i.e., to generate the regression curve, the most successful temperature-modulation and algorithm combination was the model resulting from integrating the HP-301 and HP-503 temperature profiles with the GBR algorithm. In contrast, for determining methanol concentration in commonly sold potable distilled alcohol blends, the GBR algorithm provided the best results, regardless of temperature modulation.

**Table 4:** Coefficient of determination ($\:{R}^{2}$) scores of linear regression (LR), gradient boosting regressor (GBR), stochastic gradient descent regressor (SGDR), epsilon-support vector regression (SVR), and Bayesian ridge regression (BR) models across the selected heater profiles: HP-301, HP-354, HP-503, and HP-413. Higher $\:{R}^{2}$ values indicate better performance.

	Heater profile	LR	GBR	SGDR	SVR	BR
Pure alc.	HP-301	0.997	1	0.994	0.955	0.997
	HP-354	0.997	1	0.991	0.992	0.997
	HP-503	0.998	1	0.996	0.994	0.998
	HP-413	0.999	1	0.997	0.987	0.999
Raki	HP-301	0.999	1	0.992	0.999	0.999
	HP-354	0.998	1	0.991	0.999	0.998
	HP-503	0.999	1	0.994	0.999	0.999
	HP-413	0.999	1	0.993	0.999	0.999
Whisky	HP-301	0.998	1	0.992	1	0.998
	HP-354	0.999	1	0.992	1	0.999
	HP-503	0.999	1	0.997	1	0.999
	HP-413	1	1	0.996	0.999	1

## Conclusions

This study demonstrates that the BME688 gas sensor can detect the presence of methanol and quantify its concentration in distilled potable alcohol. The results show that, when gas-composition electrical-resistance data from the BME688 sensor are combined with appropriate preprocessing techniques and machine-learning algorithms, the resulting predictions are highly accurate and reliable. However, this approach is better suited to serve as a screening layer for early warning and preliminary risk assessment, rather than as a replacement for confirmatory reference laboratory methods.

It is important to emphasise that established analytical techniques such as gas chromatography (GC), spectrophotometric methods, and enzymatic assays remain the gold standard for methanol quantification due to their high sensitivity and analytical reliability. Nevertheless, these methods require laboratory infrastructure, trained personnel, and relatively long analysis times, which limit their applicability to rapid, on-site screening. In contrast, the proposed BME688-based framework enables rapid on-site screening with significantly reduced cost, size, and operational complexity. Therefore, the primary contribution of this study lies not in competing with laboratory-grade precision but in providing a practical, deployable early-warning system that can pre-screen and prioritise samples for further confirmatory analysis.

Given that even small amounts of methanol are highly toxic to humans, potentially leading to irreversible health damage, coma, or death, its rapid and reliable detection in alcoholic beverages is of critical importance. As a low-cost, lightweight, and low-power sensing platform, the BME688-based system can be effectively deployed in alcohol production facilities and along supply chains to enable early-stage screening and continuous monitoring. This approach may help reduce methanol-related health risks while also lowering operational costs by minimising reliance on expensive laboratory equipment and controlled testing environments.

Beyond its immediate applicability, this study highlights the broader potential of integrating low-cost gas sensors with machine learning techniques for chemical safety monitoring. The strong performance observed in both classification and regression tasks suggests that such sensor–algorithm combinations may provide scalable and adaptable solutions for real-world quality-control applications. In particular, the ability not only to detect methanol but also to estimate its concentration enhances the system’s practical utility, enabling more informed decision-making in safety-critical contexts.

Nevertheless, several limitations should be acknowledged. The experiments were conducted under highly controlled laboratory conditions and involved a limited number of alcoholic beverage samples. As such, the generalizability of the results to real-world settings, where temperature, humidity, background gases, and beverage compositions may vary significantly, remains to be fully established. Future work should therefore focus on validating the proposed approach across a broader range of alcoholic beverages, methanol concentrations, and environmental conditions, and on assessing long-term sensor stability and robustness. Additionally, while the present study focused on concentration ranges of direct toxicological relevance, formally characterising the analytical limit of detection (LOD) at sub-threshold methanol concentrations represents an important direction for future work.

In future studies, integrating additional sensor modalities, adaptive calibration strategies, and advanced learning frameworks such as domain adaptation or online learning may further enhance system performance and resilience. Such developments could enable practical, deployable methanol detection systems that meaningfully contribute to public health protection and food safety assurance. Preliminary model conversion performed in this study confirms that the trained SVM model can be deployed on an ESP32 microcontroller with minimal memory and computational requirements. These results support the feasibility of developing a fully portable, battery-operated methanol screening device in future work.

## Supplementary Information

Below is the link to the electronic supplementary material.

Supplementary Material 1

Supplementary Material 2

## Data Availability

The data and source code described and used in this article are available and can be accessed at [https://github.com/ihpar/methanol](https:/github.com/ihpar/methanol) .
