# Supplementary material for: Rapid on-site methanol screening in distilled spirits via BME688 MOX sensor array and machine learning
Source: Sci Rep. 2026 Apr 25;16:19129. doi: 10.1038/s41598-026-49507-x (PMC13280137; doi:10.1038/s41598-026-49507-x)
Supplement: Supplementary file 2 — Supplementary Material 2 [file 41598_2026_49507_MOESM2_ESM.docx]

**Real-Time Methanol Detection in Distilled Spirits via BME688 MOX Sensor Array and Machine Learning**

**Highlights**

- BME688 MOX sensor matrix + ML enables real-time methanol screening in spirits.
- Measurements cover ethanol–methanol mixtures and real raki/whisky beverage matrices.
- Signal pipeline uses spline alignment plus log-transform and standard scaling for stability.
- SVM delivered 100% classification accuracy across heater profiles (HP-301/354/503/413).
- GBR achieved MAE = 0 and R² ≈ 1 for methanol quantification (best with HP-301/HP-503).
