# Supplementary material for: Rapid on-site methanol screening in distilled spirits via BME688 MOX sensor array and machine learning
Source: Sci Rep. 2026 Apr 25;16:19129. doi: 10.1038/s41598-026-49507-x (PMC13280137; doi:10.1038/s41598-026-49507-x)
Supplement: Supplementary file 1 — Supplementary Material 1 [file 41598_2026_49507_MOESM1_ESM.docx]

Dear Editor-in-Chief,

We are pleased to submit our manuscript entitled “Real-Time Methanol Detection in Distilled Spirits via BME688 MOX Sensor Array and Machine Learning” for consideration for publication in *Scientific Reports*.

In this study, we propose a rapid, low-cost, and non-destructive sensing framework for methanol detection in alcoholic beverages, using a BME688 metal-oxide (MOX) gas sensor combined with machine-learning models. The proposed approach integrates temperature-modulated sensor operation, signal preprocessing, and supervised learning algorithms to enable both classification and quantification of methanol in ethanol–methanol mixtures and real alcoholic beverage samples.

Unlike conventional laboratory-based analytical techniques, the presented system is designed for fast screening applications and practical deployment. Experimental results demonstrate that support vector machine and gradient boosting regression models achieve high classification accuracy and near-perfect quantification performance across different heater profiles. The findings highlight the potential of compact MOX sensor platforms for reliable chemical sensing in food safety and quality monitoring applications.

The manuscript is original, has not been published previously, is not under consideration for publication elsewhere, and has a 6% Turnitin similarity score. All authors have approved the manuscript and agree with its submission to *Scientific Reports*. No conflicts of interest are declared.

We believe that the methodological focus on sensor signal processing, model evaluation, and measurement reliability aligns well with the scope and readership of *Scientific Reports*. We appreciate your time and consideration, and we look forward to your response.

Sincerely,

İsmail Hakkı Parlak
Bolu Abant İzzet Baysal University
Computer Engineering Department
Bolu, Turkey
ismail.parlak@ibu.edu.tr
